# Flow Injection Spectrophotometric Determination of *N*-Acetyl-l-cysteine as a Complex with Palladium(II)

**DOI:** 10.3390/molecules16097224

**Published:** 2011-08-25

**Authors:** Josipa Giljanović, Mia Brkljača, Ante Prkić

**Affiliations:** 1 Department of Analytical Chemistry, Faculty of Chemistry and Technology, Teslina 10/V, 21000 Split, Croatia; 2 Department of Mediterranean Agriculture and Aquaculture, University of Zadar, Mihovila Pavlinovića bb, 23000 Zadar, Croatia; E-Mail: mbrkljaca@unizd.hr

**Keywords:** flow-injection, spectrophotometry, *N*-Acetyl-l-cysteine, palladium, complexometry

## Abstract

We describe a new method using flow-injection analysis with spectro-photometric detection, suitable for the determination of *N*-acetyl-l-cysteine (NAC). The proposed method is appropriate for the determination of NAC in reaction with Pd^2+^ ions in the concentration range from 1.0 × 10^−5^ mol L^−1^ to 6.0 × 10^−5^ mol L^−1^. The detection limit NAC was 5.84 × 10^−6^ mol L^−1^ and the recorded relative standard deviation of the method is in the range from 1.67 to 4.11%. NAC and Pd^2+^ form complexes of Pd^2+^:NAC molar ratios of 1:1 and 1:2, depending on the ratio of their analytical concentrations. The cumulative conditional stability constant for the Pd(NAC)_2_^2+^ complex is *β*_12_’ = 2.69 × 10^9^ L^2^ mol^−2^. The proposed method was compared with the classic spectrophotometric determination of NAC, using the same reagent, PdCl_2_, and had shown certain advantages: a) shorter analysis time; b) the use of smaller volumes of sample and reagents, which make the proposed method cheaper and faster for NAC determination in real samples without sample pretreatment.

## 1. Introduction

*N*-acetyl-l-cysteine, NAC, is a biologically active substance used as mucolitic agent and as an antidote against paracetamol poisoning. NAC has been determined by direct potentiometry [1,2,3,4,5], different Flow-injection analysis (FIA) methods (potentiometry [6], indirect FIA [7], chemiluminescence-FIA [8]), spectrophotometry [9,10,11,12], spectrophotometric catalytic titration [13,14], enzyme spectrophotometry [14], LC-UV-MS [15] and different chromatographic techniques [16,17,18,19].

Several NAC determination methods by spectrophotometry are described based on formation of the colored compounds using different substances: Fe^3+^ and ferrozine [9], *o*-phthalaldehyde and isoleucine [10], or palladium(II) [11]. Penicillamine and cysteine have been determined by FIA spectrophotometry [12]. The NAC complex with palladium(II) has been determined by spectrophotometry in large volumes [11]. Since flow injection determinations have advantages in speed, precision and lower chemical consumption [20], flow-injection analysis using Pd^2+^ for determination of NAC was applied.

## 2. Results and Discussion

*N*-acetyl-l-cysteine reacts with Pd^2+^ to form a yellow complex; this reaction has been used by Jovanović and Stanković [11] for the spectrophotometric determination of injection formulations. At the chosen experimental conditions large volume spectrophotometric analyses were conducted in order to establish the stoichiometric ratio of the complex and conditional stability constants.

**Figure 1:**
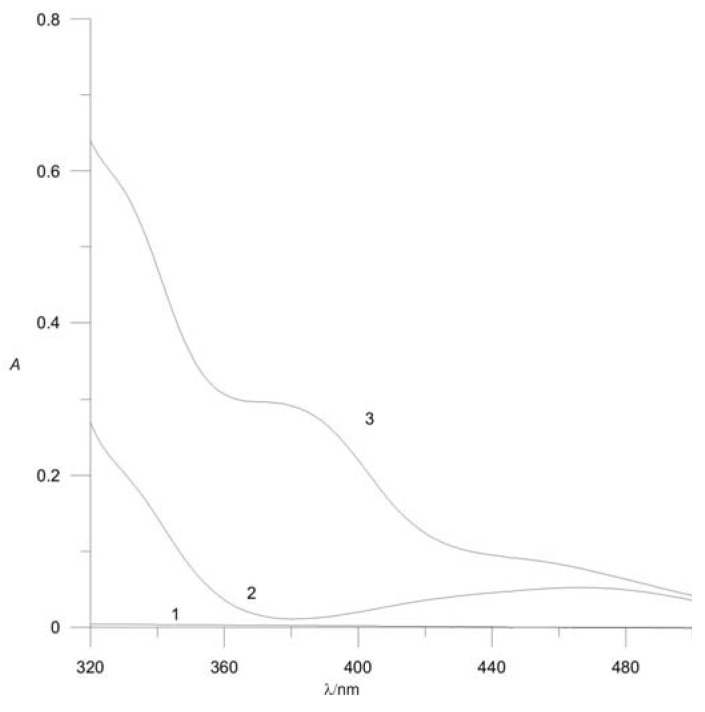
Absorption spectra of: **(1)** N-Acetyl-*l*-cysteine; **(2)** PdCl_2_; **(3)** the NAC-Pd^2+^ complex. [NAC] = 2 × 10^−4^ mol L^−1^; [Pd^2+^] = 5 × 10^−4^ mol L^−1^; pH = 3; *μ* = 0.2 mol L^−1^.

Absorption spectra were recorded over the range 250-700 nm (Figure 1). The complex shows a maximum absorbance at 380 nm, which was therefore used as the wavelength for the determination. At this wavelength *N*-Acetyl-l-cysteine does not absorb and palladium(II) chloride has a low absorbance. All measurements were performed against a reagent blank.

**Figure 2:**
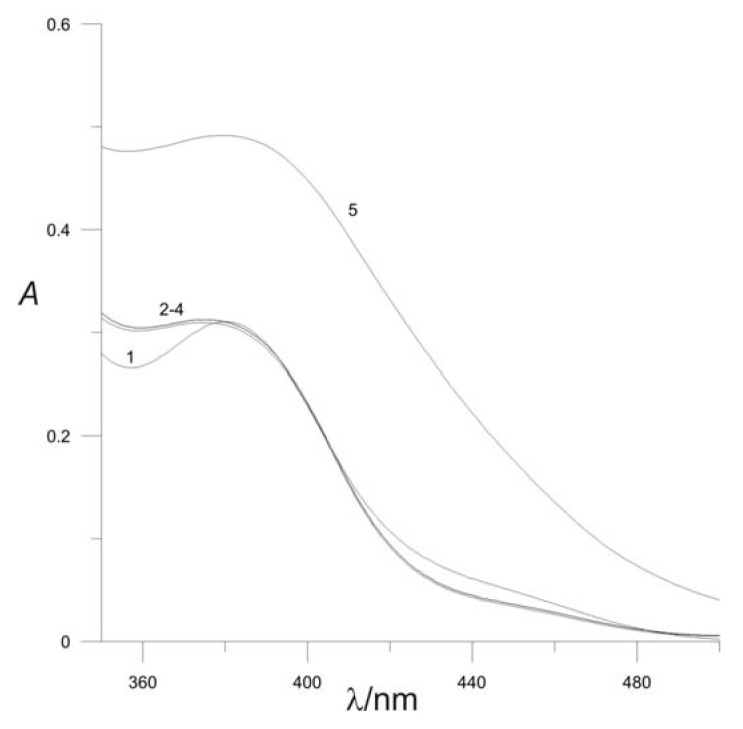
Effect of pH on complex formation: [NAC] = 2 × 10^−4^ M; [Pd^2+^] = 5 × 10^−4^ mol L^−1^; pH = 3; *μ* = 0.2 mol L^−1^. **(1)** pH = 1.96; **(2)** pH = 3.52; **(3)** pH = 4.62; **(4)** pH = 5.33; **(5)** pH = 7.00.

Influence of pH of the reaction mixture on the amounts of NAC-PdCl_2_ complex produced is shown on the Figure 2. 

The absorbance is similar in the pH range 1.96 to 4.62 and increases at pH 7.00. The probable reason for the rising absorbance of the formed complex at pH = 7 is that by increasing the pH value, the dominant NAC species becomes a deprotonated one. Eventually, the deprotonated NAC species forms a complex with Pd^2+^. The best NAC - PdCl_2_ complex spectra were obtained at pH 2. The shape of the absorption spectrum and the position of the maximum of the NAC - PdCl_2_ complex do not vary with pH, which indicates that under experimental conditions only one type of complex is formed.

**Figure 3:**
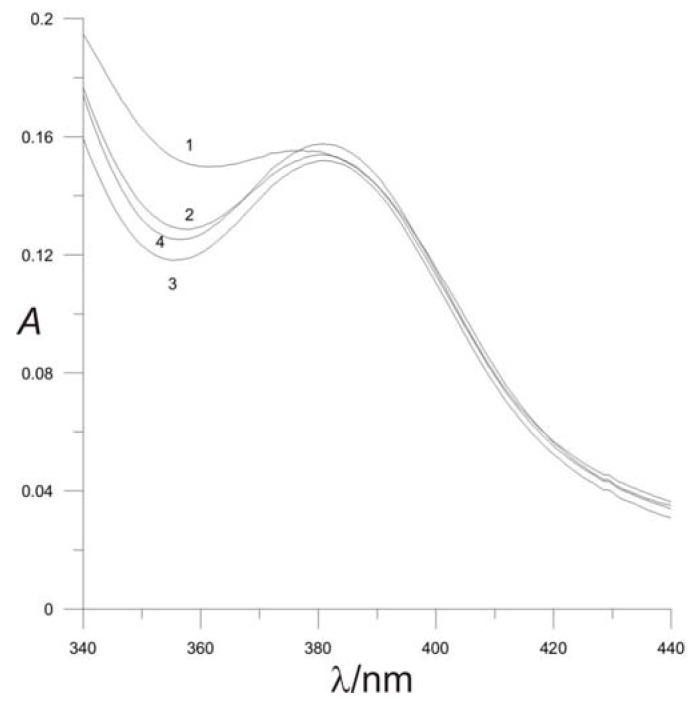
Effect of ionic strength on complex formation: [NAC] = 1 × 10^−4^ mol L^−1^; [Pd^2+^] = 5 × 10^−4^ mol L^−1^; pH = 2, **(1)** *μ* = 0.1 mol L^−1^; **(2)** *μ* = 0.4 mol L^−1^; **(3)** *μ* = 0.6 mol L^−1^, **(4)** *μ* = 0.8 mol L^−1^.

The ionic strength has little influence on the course of reaction. Different ionic strengths were adjusted by adding the needed mass of solid NaCl and dissolving in solution of the desired pH value. The best spectrum was obtained at an ionic strength of 0.4 mol L^−1^ (Figure 3).

**Figure 4:**
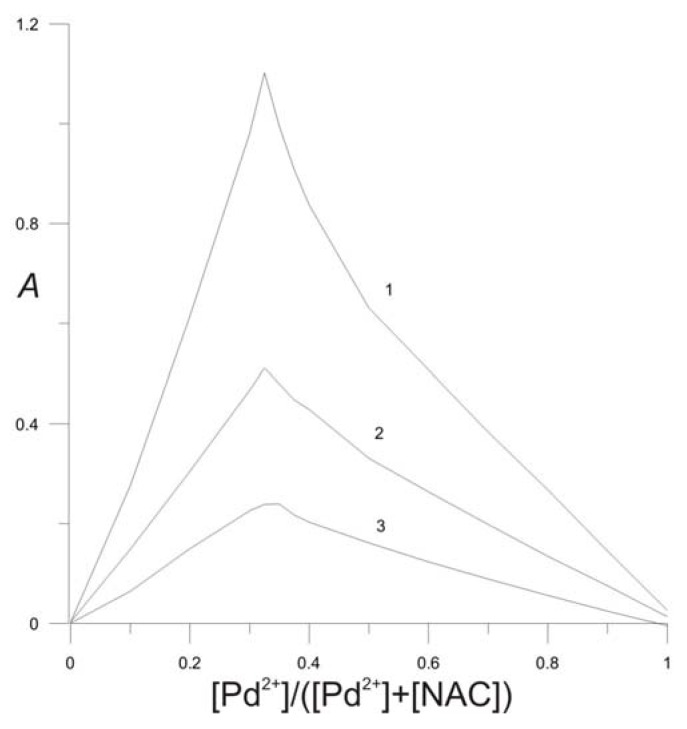
Job’s plot for isomolar solution series: **(1)** [Pd^2+^] + [NAC] = 8 × 10^−4^ mol L^−1^; **(2)** [Pd^2+^] + [NAC] = 4 × 10^−4^ mol L^−1^; **(3)** [Pd] + [NAC] = 2 × 10^−4^ mol L^−1^; pH = 2; *μ* = 0.4 mol L^−1^.

**Figure 5:**
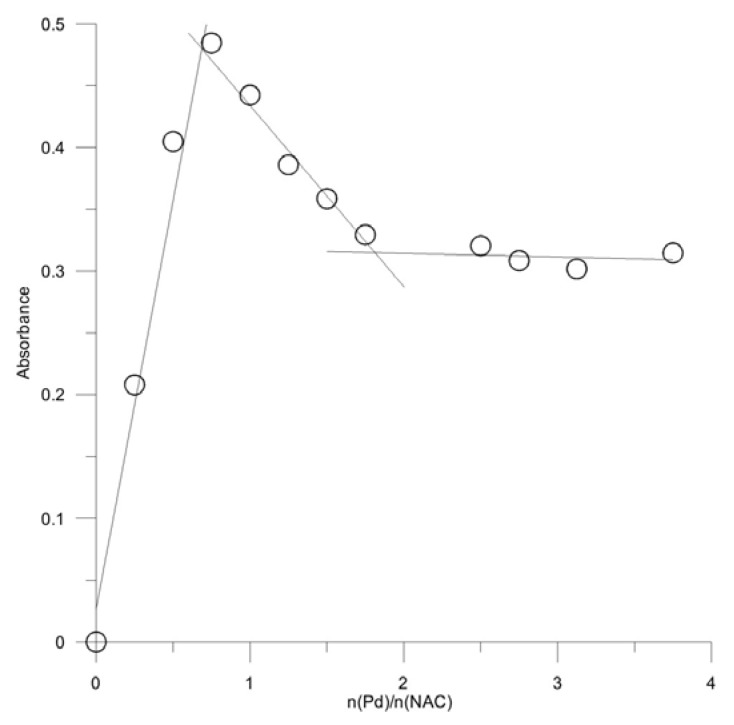
Molar ratio method: [NAC] = 4 × 10^−4^ mol L^−1^; pH = 2; *μ* = 0.4 mol L^−1^.

The stoichiometric ratio of NAC to Pd^2+^ in the complex was determined by Job’s continuous variation method (Figure 4). Each of the three curves for three isomolar solution series reaches a single maximum value corresponding to the same mole fraction, [Pd^2+^]/([Pd^2+^] + [NAC]), of 0.33, which proves that a single complex compound was formed and that its Pd^2+^:NAC molar ratio = 1:2. The results were confirmed by the molar ratio method. The plot obtained at constant [NAC] had a maximum value at molar ratio of Pd^2+^ to NAC of 0.50, which indicated the formation of complex Pd^2+^ to NAC molar ratio of 1:2.0 (Figure 5). The conditional stability constant *K*’ of the Pd-NAC complex was calculated by using data of the methods of molar ratios and of the method of continuous variation (Table 1). 

At maximum absorbance the concentration of the PdCl_2_ - NAC complex is equal to analytical concentration of PdCl_2_ in that point:

(1) $$A_{\max}=c\left({\mathrm{Pd}}^{2+}\right)\cdot a_{{\mathrm{Pd}(\mathrm{NAC})}_{2}}\cdot b\;$$

In every solution *i* the concentration of complex Pd^2+^-NAC is:

(2) $$A_{i}={\left[{\mathrm{Pd}(\mathrm{NAC})}_{2}\right]}_{i}\cdot a_{{\mathrm{Pd}(\mathrm{NAC})}_{2}}\cdot b\;$$

From relations (1) and (2) [Pd(NAC)_2_]_i_ is calculated. The [Pd^2+^]_i_ and [NAC]_i_ concentrations are calculated from Equations (3) and (4):

(3) $${\left[{\mathrm{Pd}}^{2+}\right]}_{i}=c\left({\mathrm{Pd}}^{2+}\right)-{\left[{\mathrm{Pd}(\mathrm{NAC})}_{2}\right]}_{i}\;$$

(4) $${\left[\mathrm{NAC}\right]}_{i}=c(\mathrm{NAC})-2\cdot {\left[{\mathrm{Pd}(\mathrm{NAC})}_{2}\right]}_{i}\;$$

The cumulative conditional stability constants, *β*_2_, are calculated using calculated equilibrium concentrations of Pd(NAC)_2_^2+^, Pd^2+^ and NAC and using Equation (5):

(5) $${\beta_{i}}^{\prime }=\frac{{\left[{\mathrm{Pd}(\mathrm{NAC})}_{2}^{2+}\right]}_{i}}{{\left[{\mathrm{Pd}}^{2+}\right]}_{i}\cdot {{\left[\mathrm{NAC}\right]}_{i}}^{2}}\;$$

The results are presented in Table 1.

Beer’s law was verified in buffer solution at pH 2 (Figure 5). 

A linear relationship between the absorbance and the concentration of NAC was obtained over the range 1.0 × 10^−5^ to 6.0 × 10^−4^ M NAC. The regression equation was *A* = 0.1649 × *c*(NAC) − 0.0166, with a correlation coefficient of 0.9980 (*n* = 8), indicating good linearity.

### 2.1. Flow-Injection Analysis

**Figure 7:**
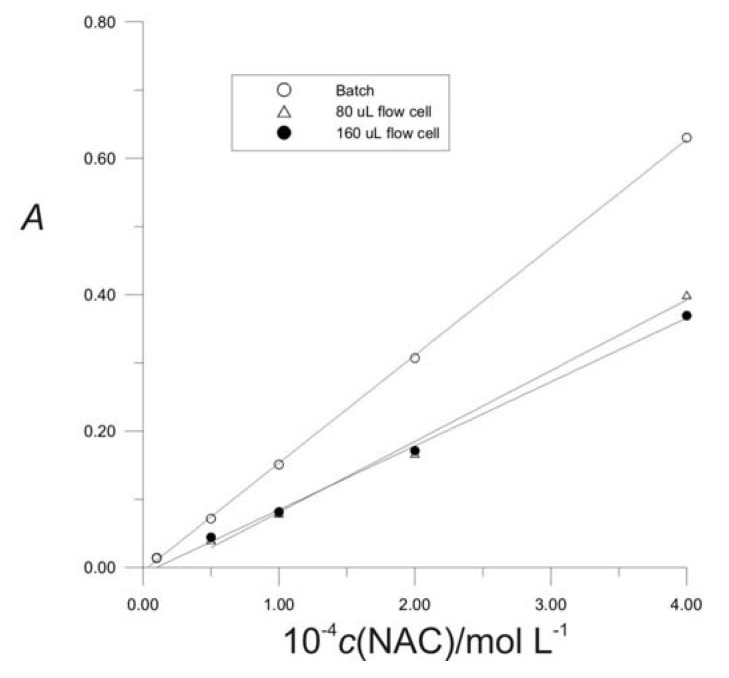
Effect of flow cell volume on peak height. Injection loop, *V* = 1000 μL, pumping rate *Q* = 7.5 mL min^−1^.

Since the chemical reaction is fast, an equilibrium method can be easily adopted to the FIA technique using the simple manifold as shown in Figure 9. For the analyses a flow cell volume of 80 μL was chosen since calibration curve at 80-μL flow cell was closer to the curve of stationary state than calibration curve of a 160-μL flow cell (Figure 7).

**Figure 8:**
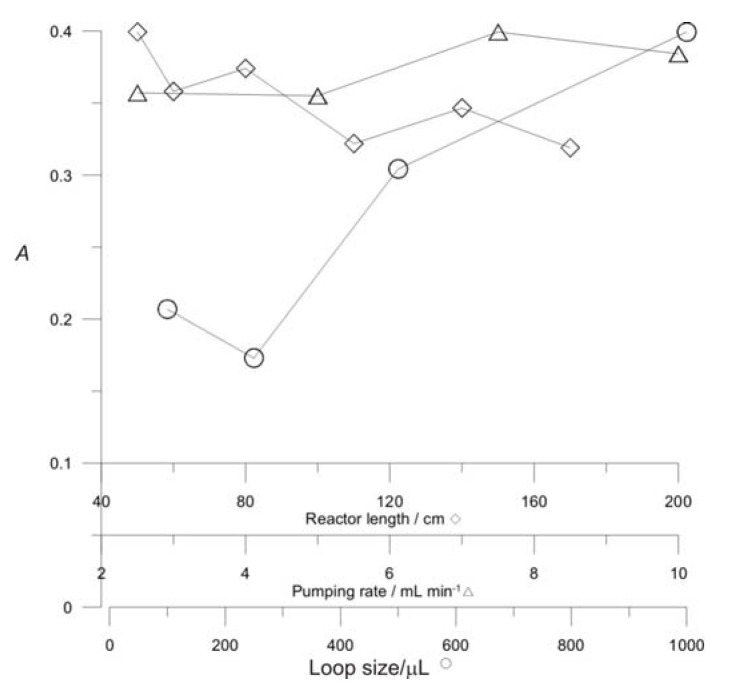
Effect of loop size: **(a)**, pumping rate; **(b)** reactor length; **(c)** on the peak height. Sample injected 4.0 × 10^−4^ mol L^−1^ NAC.

**Figure 9:**
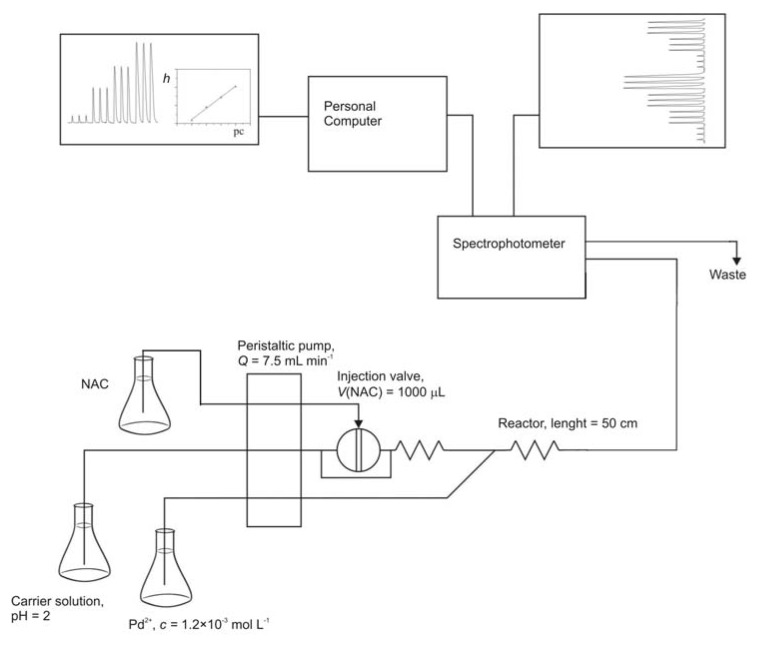
FIA manifold for the determination of *N*-acetyl-l-cysteine.

Figure 8 shows the effect of the loop size, coil length, and pumping rate on the peak height. An increase in loop size produces an increase in peak height (Figure 8a); when the volume of NAC injected is 1000 μL the absorbance is high providing relatively high sample throughput of 70 samples per hour. Thus, a 1000 μL loop size was chosen. The pumping rate was increased from 2.5 to 10 mL min−1 (Figure 8b). The maximum absorbance values were at 7.5 mL min^−1^. Thus, a 7.5 mL min^−1^ pumping rate was selected. The influence of the coil length was studied between minimum possible distance between the mixing point and the detector and 1.70 m (Figure 8c). The results showed that the peak height continuously decreases with the increase of the reactor length. The complexation reaction occurs instantaneously and by increasing the length of the coil, the dispersion will be higher. Thus, the coil has to be as short as possible, to avoid dispersion effects.

In order to find the optimal concentration of Pd^2+^ in the reagent solution, the concentration of PdCl_2_ was varied from 1.0 × 10^−5^ to 1.5 × 10^−3^ M. A constant absorbance was achieved for total molar ratios of Pd^2+^ to NAC higher than 2:1. Thus, the concentration of PdCl_2_ of 1.2 × 10^−3^ M was chosen.

The optimal values for the FIA parameters were as follows: injected volume 1,000 μL; reactor length 50 cm, pumping rate 7.5 mL min^−1^ and PdCl_2_ concentration 1.2 × 10^−3^ M. For the described manifold, the calibration graph for the determination of *N*-acetyl-l-cysteine is linear between 1.0 × 10^−5^ and 6.0 × 10^−4^ M. The regression equation was y = 0.008265 × x + 0.011802, with a correlation coefficient of 0.9976. The detection limit [21] was 5.84 × 10^−5^ M NAC, which was determined by measuring absorbance of the carrier solution and using Equation (6):

(6) $$c_{L}=\frac{3\cdot s_{B}}{a}-b\;$$

where *a* and *b* are slope and intercept of the calibration curve, *n* = 20.

Comparison of our FIA method to the equilibrium method for Pd–NAC determination by spectrophotometry is shown in Table 2. Although the equilibrium method uses a lower concentration of PdCl_2_, the FIA method has the advantage over the equilibrium method of significantly higher sample throughput.

In Table 3 are given data for the calculated conditional constants of complexes between NAC and Pd^2+^ in acidic medium at pH = 2 and 25 °C and their compared with conditional constants of complexes between NAC and other transition metals. The palladium and NAC complex has medium stability according to this data.

Table 4 gives a comparison of analytical parameters of three different NAC determination techniques, two of which are FIA methods. The method proposed by Jovanović and Stanković [11] is a classic spectrophotometric analysis and it is very slow in comparison with the FIA methods and can be hardly compared with these. The method of Jovanović and Stanković has a shorter linear response range and greater limit of detection (LOD) in comparing with the FIA methods. 

When the FIA method proposed by Sanchez-Pedreño *et al.* [23] is compared with our proposed FIA method, it can be seen that our method has a lower limit of detection (LOD), works in mildly acidic medium (pH = 2) and the linear response range begins at lower NAC concentrations.

It has also been shown that the proposed FIA method is simple, rapid and accurate and can be applied to the determination of NAC in Fluimukan injections. The precision of determination was studied using three Fluimukan injection samples. The applicability of the method for the assay of simple dosage forms was examined by analyzing Fluimukan injections. The relative standard deviation (RSD) of the method was from 1.67 to 4.11% (n = 5, Table 5). The determination of NAC in Fluimukan samples was performed without any pharmaceutical pretreatment.

## 3. Experimental

### 3.1. Apparatus

A Shimadzu 1601-UV spectrophotometer with 10-mm quartz cells was used for recording spectra and absorbance measurements. The flow injection system consisted of a ISMATEC IPC peristaltic pump, injection valve (model V-100, Tecator, Sweden), a Hellma 80 μL and 160 μL flow cells and the Shimadzu 1601-UV spectrophotometer as detector. Connecting tubing and connectors were 0.5 mm i.d. (Rheodyne, USA).

### 3.2. Reagents

All chemicals were of analytical grade and dissolved in suprapure water made by a Millipore Simplicity system (Millipore, USA). Palladium chloride was supplied by Merck (Germany), sodium hydroxide, hydrochloride acid, acetic acid and phosphorus acid from Kemika (Croatia) were used. *N*-Acetyl-l-cysteine was supplied by Merck. Fluimukan injections from Lek Ljubljana (Slovenia) were used for testing the method.

Buffer solution, pH = 2, which also used as carrier solution in FIA, was prepared by dissolving acetic acid (4.7980 g), boric acid (4.9464 g) and phosphorus acid (5.4580 g) in suprapure water.

Solution for equilibrium measurements of stability constant and stoichiometric ratio was prepared in a 25-mL volumetric flask by adding appropriate volumes of *N*-acetyl-l-cysteine and PdCl_2_ solutions and diluting to the mark with carrier solution. The solutions’ absorbencies were measured as absorbance heights at 380 nm against reagent blank.

FIA measurements were conducted using the simple manifold shown in Figure 1. The carrier solution was prepared by adding NaCl (2.338 g) for constant ionic strength (*μ*) and buffer pH = 2 (400 mL) to a 1-L volumetric flask, and diluting to the mark with suprapure water. The carrier solution ionic strength was adjusted with 0.4 mol L^−1^ NaCl. The reagent solution was prepared by adding PdCl_2_ (0.1068 g), a few drops of concentrated HCl and approximately 400 mL of suprapure water to a 500-mL volumetric flask. The flask was placed in a warm water bath or left overnight to dissolve. Solution was diluted to the mark with suprapure water. The reagent solution was 1.2 × 10^−3^ mol L^−1^ PdCl_2_. The calibration graph was prepared over the range 1 × 10^−5^ mol L^−1^ – 6 × 10^−4^ mol L^−1^ NAC. Sample volume injected was 1000 μL and solutions absorbencies were measured as the peak height at 380 nm.

## 4. Conclusions

A new method using flow-injection analysis with spectrophotometric detection suitable for the determination of *N*-acetyl-l-cysteine (NAC) was described. NAC and Pd^2+^ form complexes of molar ratio of Pd^2+^:NAC 1:1 and 1:2, depending on the ratio of their analytical concentrations. The average conditional stability constant for the Pd(NAC)^2+^ complex is *K*_1_’= 6.61 × 10^5^ L mol^−1^ and for the Pd(NAC)_2_^2+^ complex is *K*_2_’ = 4.07 × 10^3^ L^2^ mol^−2^. Molar absorption coefficient for Pd(NAC)^2+^ is 1,599.8 ± 36, *n* = 20 and for Pd(NAC)_2_^2+^ is 3,875.6 ± 23, *n* = 20. The NAC detection limit is 5.84 × 10^−6^ mol L^−1^ and relative standard deviation of the method ranges from 1.67 to 4.11% for determination of NAC in real samples without pretreatment. The proposed method is appropriate for determination of NAC in the concentration range from 1.0 × 10^−5^ mol L^−1^ to 6.0 × 10^−4^ mol L^−1^ in reaction with Pd^2+^ ions. The proposed method was compared with the classic spectrophotometric determination of NAC, using the same reagent, PdCl_2_, and had shown certain advantages in a shorter time of analysis, and the use of smaller volumes of sample and reagents, which means the proposed method for NAC determination in real samples is cheaper and faster without sample pretreatment.

## Figures and Tables

1 - molar ratios method at constant [Pd^2+^] of 1.0 × 10^−4^ M; 2 - molar ratios method at constant [NAC] of 4.0 × 10^−4^ M; 3 - continuous variation method, isomolar solution series 1; 4 - continuous variation method, isomolar solution series 2.

**Figure 6:**
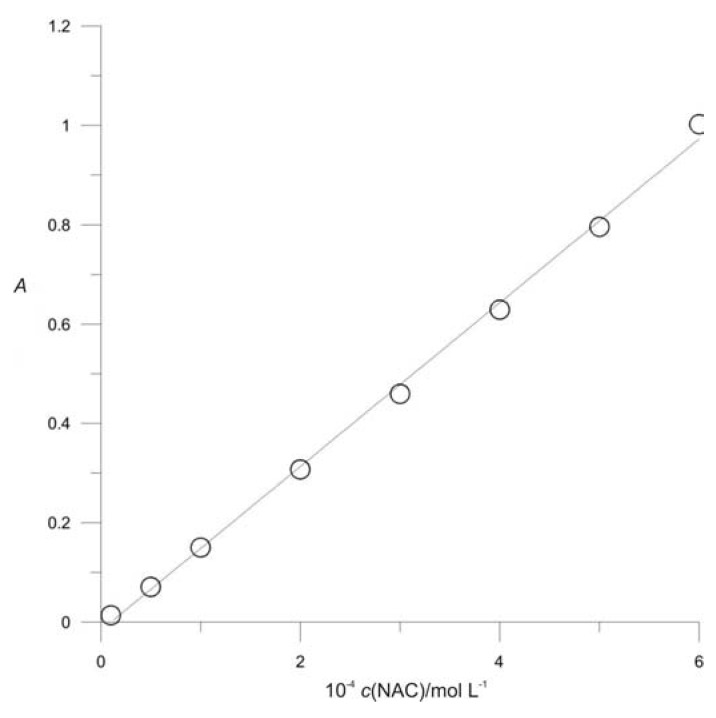
Calibration curve. [Pd^2+^] = 5 × 10^−4^ M, pH = 2, *μ* = 0.4 mol L^−1^.

**Table 1:** Cumulative conditional stability constant (*β*’) of the Pd(NAC)_2_^2+^ complex. Conditions: pH = 2; *μ* = 0.4 mol L^−1^.

log$\bar{K^{\prime }}$ = log*β*_2_	Method
9.01	1
8.13	2
8.11	3
8.45	4
8.43, RSD = 4.98%	log $\bar{K^{\prime }}$

**Table 2:** Comparison of analytical parameters for two methods of NAC determination.

Parameter	Equilibrium method	FIA method
calibration range	1 × 10^−5^ – 6 × 10^−4^ mol L^−1^ NAC	1 × 10^−5^ – 6 × 10^−4^ mol L^−1^ NAC
Molar absorption coefficient, *ε* / L mol^−1^ cm^−1^	Pd(NAC)^2+^: 1599.8 ± 36, *n* = 20 Pd(NAC)_2_^2+^: 3875.6 ± 23, *n* = 20	
coefficient of determination, *R*^2^	0.9980	0.9976
PdCl_2_ concentration	5.0 × 10^−4^ mol L^−1^	1.2 × 10^−3^ mol L^−1^
sample throughput per hour	30	70

**Table 3:** Conditional constants of forming complexes between NAC and different metals.

Metal	log*K*_1_	log*K*_2_	log*β*_2_
Cr^3+ a^	8.26	6.74	15.00
Co^2+ a^	4.18	3.62	7.80
Ni^2+ a^	5.02	4.32	9.34
Fe^3+ a^	10.58	8.22	18.80
Pd^2+ b^	4.82	3.61	8.43

^a^ *μ* = 0.12 mol L^−1^ (NaClO_4_), *t* = 25 °C^22^; ^b^ this work

**Table 4:** Comparison of analytic parameters for three different techniques for NAC determination.

Analytical parameters	Classic Spectrophotometric analysis [11]	FIA Spectrophotometric analysis [23]	FIA Spectrophotometric analysis ^a^
Linear response range, mol L^−1^ NAC	2.45 × 10^−5^ – 4 × 10^−4^	5 × 10^−5^ – 5 × 10^−3^	1 × 10^−5^ – 6 × 10^−4^
Correlation factor (R)	0.9970	0.9995	0.9980
*μ*, mol L^−1^	0.2	1.0	0.4
pH	4.64	0	2
LOD, mol L^−1^	2.0 × 10^−5^	1.6 × 10^−5^	5.84 × 10^−6^
Reproducibility (RSD) / %	0.63 – 1.92	1.4, *n* = 10	1.04 – 1.22, *n* = 10
Recovery (RSD) / %	0.70, *n* = 7	N/A	1.67 – 4.11, *n* = 3

**Table 5:** Determination of NAC in real samples.

	Labeled, mg	Found ±SD (%) (*n* = 5)	Recovery (%)
*Fluimukan*®	100	101.2 ± 4.11	101.23
	200	203.2 ± 2.54	101.60
	600	603.3 ± 1.67	100.55
